# Instrument for Exhibiting the Motions of the Chest during Respiration

**Published:** 1847-10

**Authors:** 


*Instrument for exhibiting the motions of the Chest during Respiration.*—Mr. Sibson, of Nottingham, exhibited at a meeting of the Provincial Medical Association a newly-invented instrument, for the purpose of ascertaining the comparative movements of the chest in respiration. The instrument, which was of simple construction, consisted of a dial-plate, on which were indicated degrees of the one-hundreth part of an inch, attached to which was a rack and pinion ; as the rack rose, by the expansion of the chest, the pinion, which ran through, communicated with the indicator, which showed upon the dial-plate the number of degrees the chest rose, and the rack was returned by means of a spring. If the instrument was held steadily on the chest, it would show the precise amount of its expansion. Where he had the privilege, as he had at the hospital to which he was attached, he preferred his patients should be in bed to test the chest, though it could be done without, as the instrument would show the workings of the chest when a person was either sitting or standing. If he had any of the gentlemen he was then addressing with him at the bedside of a patient, they would delight in the accuracy of the working of the instrument. He was first assisted in the design of it by an operative, who was a patient in the hospital at Nottingham ; but the perfect instrument which he was then shewing them, was made by Mr. Simmonds, an eminent watchmaker of London, to whom great credit was due for the accuracy of its construction. With regard to the movements of the chest in health and disease, he would remark, that out of ten patients who supposed themselves to be suffering from disease in the chest, in nine of them perhaps it was nothing of the sort, and with the assistance of that little instrument, or the spirometer of Dr. Hutchinson, which had been exhibited to them by Dr. Shearman, they could send their patients home with the pleasing fact upon their minds that they were healthy men. Mr. Sibson then went on to show that, by the instrument before the meeting, he could more accurately trace the seat of the disease in the chest, and ascertain whether the disease was seated in the upper or lower lobe of the lungs, or any part of them, as the instrument indicated the contraction and expansion caused by expiration and inspiration, and would also show the movement of the abdomen. In healthy subjects the times of inspiration and expiration were generally the same, though in many subjects there would be a pause at the conclusion of inspiration. Mr. Sibson then exemplified the working of the instrument, the accuracy of which was fully acknowleged, on his own chest, and explained that a difference of three degrees in the movement of one side of the chest as compared with the other, when indicated by the instrument, was sufficient to cause attention to be turned towards it, though it did not necessarily follow that where there was such difference, the lungs might be diseased, as abscess of the ribs, or any disease or injury of them, might cause one side of the chest to move less liberally than the other ; so that they must not always conclude that the lungs were affected when they discovered this difference, as in one instance he knew, it had been caused by a diseased shoulder. He compared the instrument to a pigmy spirometer, capable of being carried in the pocket of a medical man, who, when he had to travel upon horseback, could not carry with him the valuable instrument of Mr. Hutchinson.

An improved tube, used in laryngotomy, was also exhibited by Mr. Sibson ; and a simple and novel construction to supersede artificial inspiration by means of bellows, in cases of drowning, &c. The latter consisted of a piece of flat flexible metal, with a handle at the back, and covered on the face of it with a newly-discovered adhesive composition; this being placed on the chest and gently drawn upwards, and compressed downwards, so as to carry the walls of the chest with it, would, he thought, supersede the use of the bellows in many cases. At the same time he must deprecate the pressure upon the bowels used in cases of drowning or poisoning sometimes, as he had frequently found from *post-mortem* examination such pressure was highly injurious.—*Prov. Med. and Sur. Jour.*

